# Parliament and the Professions

**Published:** 1921-01-01

**Authors:** 


### Parliament and the Professions.

### Uncertified Deaths.

The Minister of Health has stated that the number of uncertified deaths reported in 1919 by a registrar of births and deaths to a coroner which were not the subject of an inquest was 6,698. The total number of inquests held in that year were 31,488, but no figures are available as to how many of these related respectively to certified and uncertified deaths.

### Salvarsan Substitutes.

The Minister of Health stated that the following substitutes for salvarsan and neo-salvarsan have been approved: Kharsivan, neo-kharsivan, arsenobillon, lovarsenobillon, diarsenol, neo-diarsenol, galyl, and salvarsan (manufactured by an English firm of chemists). No guarantee of the Ministry that the substitutes are exactly similar to the German preparations can be given, but there is good reason to believe that all these drugs, with the exception of galyl, correspond in general chemical constitution to the respective German preparations. They are all officially tested for toxicity before being placed on sale.

### Panel Medicines.

Mr. Casey called the attention of the Minister of Health to a case where a panel doctor had been surcharged £15 for prescribing too expensive medicines to panel patients. Dr. Addison said he was making inquiries, and pointed out that medical benefit under the Insurance Acts includes the provision of "proper and sufficient medicines," and no doctor can be surcharged in respect of prescribing unless the character or quantity of the drugs is "in excess of what may reasonably be necessary for the adequate treatment of his insured patients." Any local decision to surcharge is subject to appeal to the Ministry.

